# Factors distinguishing invasive from pre-invasive adenocarcinoma presenting as pure ground glass pulmonary nodules

**DOI:** 10.1186/s13014-020-01628-x

**Published:** 2020-07-31

**Authors:** Huan-Huan Yang, Yi-Lv Lv, Xing-Hai Fan, Zhi-Yong Ai, Xiu-Chun Xu, Bo Ye, Ding-Zhong Hu

**Affiliations:** 1 Department of ThoracicSurgery, Shanghai Chest Hospital, Shanghai Jiaotong University, Shanghai, 200030 China; 2 Department of Respiratory Medicine, Shanghai Zhongye Hospital, Shanghai, 200941 China

**Keywords:** Adenocarcinoma, Ground-glass nodule, Computed tomography

## Abstract

**Background:**

To investigate predictors of pathological invasiveness and prognosis of lung adenocarcinoma in patients with pure ground-glass nodules (pGGNs).

**Methods:**

Clinical and computed tomography (CT) features of invasive adenocarcinomas (IACs) and pre-IACs were retrospectively compared in 641 consecutive patients with pGGNs and confirmed lung adenocarcinomas who had undergone postoperative CT follow-up. Potential predictors of prognosis were investigated in these patients.

**Results:**

Of 659 pGGNs in 641 patients, 258 (39.1%) were adenocarcinomas in situ, 265 (40.2%) were minimally invasive adenocarcinomas, and 136 (20.6%) were IACs. Respective optimal cutoffs for age, serum carcinoembryonic antigen concentration, maximal diameter, mean diameter, and CT density for distinguishing pre-IACs from IACs were 53 years, 2.19 ng/mL, 10.78 mm, 10.09 mm, and − 582.28 Hounsfield units (HU). Univariable analysis indicated that sex, age, maximal diameter, mean diameter, CT density, and spiculation were significant predictors of lung IAC. In multivariable analysis age, maximal diameter, and CT density were significant predictors of lung IAC. During a median follow-up of 41 months no pGGN IACs recurred.

**Conclusions:**

pGGNs may be lung IACs, especially in patients aged > 55 years with lesions that are > 1 cm in diameter and exhibit CT density > − 600 HU. pGGN IACs of < 3 cm in diameter have good post-resection prognoses.

## Background

In computed tomography (CT) images pure ground-glass nodules (pGGNs) are visualized as homogeneous hazy lesions of the lung in which the vascular and bronchial components are preserved and there is no solid component. The use of CT to screen for lung cancer has resulted in detection of an increasing number of pGGNs [1], which are consistently found to constitute precursors of lung invasive adenocarcinomas (IACs). pGGNs include atypical adenomatous hyperplasia, adenocarcinoma in situ (AIS), and minimally invasive adenocarcinoma (MIA) [2–4]. These lesions often grow slowly and have good prognoses [5–8];however, many pGGNs > 1 cm in diameter eventually develop into IACs, which have a worse prognosis [9] andrequire different therapeutic strategies [10, 11]. Therefore, the ability to detect these IACs preoperatively on the basis of clinical manifestations and CT characteristics is beneficial. The purpose of the current study was to identify factors that distinguished IACs from MIAs and AISs in patients with pGGNs who had undergone surgical resection, as well as to determine their prognoses.

## Materials and methods

### Study design

This study was conducted in accordance with the Declaration of Helsinki. The Institutional Review Board and the Ethics Committee of Shanghai Chest Hospital approved the study, and waived the requirement for patient consent due to its retrospective design.

### Patients

A search of the electronic medical records of Shanghai Chest Hospital yielded 5748 consecutive patients with a pathological diagnosis of lung cancer who had undergone surgical resection at the Department of Thoracic Surgery between January 2014 and December 2015. Inclusion criteria were (1) availability of pre-resection CT; (2) lesions manifesting as pGGNs on CT; (3) lesions < 3 cm in diameter as determined via CT; and (4) pathologically proven lung adenocarcinoma. Exclusion criteria were: (1) no pre-resection CT available; (2) lesions manifesting as part-solid or solid nodules on CT; (3) lesions > 3 cm in diameter on CT; and (4) histological diagnosis other than lung adenocarcinoma, such as AAH or squamous cell carcinoma. The following clinical data were recorded for all included patients: age, sex, smoking history (never or current/former), symptoms (none, or any of cough, shortness of breath, fever, hemoptysis, chest pain, recurrent pulmonary infection), serum carcinoembryonic antigen (CEA) concentration, and CT features. All patients underwent preoperative thoracic unenhanced CT examination at our hospital.

*CT imaging* Lung CT scans were performed with a Somatom Sensation-64 (Siemens Medical Systems, Forchheim, Germany) with 120 kVp and 100 mAs. All CT examinations included the entire thorax at full suspended inspiration with the patient lying supine. When a nodule was identified, target CT was performed with the following parameters: pitch, 0.64; 1–3 s scan time; matrix size, 1024*1024; FOV, 180 mm.The lung window width was consistently 1600 Hounsfield units (HU), and the window level was − 600 HU.

Nodule size was expressed as maximal (longest diameter on axial images) and mean (average of the maximum length and width of the nodule) diameters [12]. CT density was defined as the average CT attenuation (HU) within the nodule that did not contain blood vessels or bronchioles. Two readers (radiologist J.G. with 16 years of experience in chest CT and surgeon J.F. with 12 years of experience in thoracic surgery) who were blinded to the histopathological results and clinical data evaluated all CT scans independently. Each reader measured the sizes and d of the lesions in the lung window setting on the transverse CT section that displayed the largest nodule dimensions. The average of the measurements obtained by each of the two reviewers was used for analysis. The observers also recorded the presence of particular signs such as pleural retraction, air bronchogram, bubble lucency, and spiculated margins. Pleural retraction was defined as linear attenuation heading toward the pleura or the major or minor fissure from a pGGN. Air bronchogram was defined as air-filled bronchi within a pGGN. Bubble lucency was defined as the presence of small spots of round or ovoid air attenuation within a pGGN. Spiculated margins were defined as the presence of strands extending from a nodule margin into the lung parenchyma without reaching the pleural surface [4]. The results were compared between two readers. If the results were discrepant, the two readers reevaluated the scan to reach a consensus. If no consensus was reached, another radiologist (Z.X.G. with 24 years of experience) was consulted and their decision was deemed final.

### Histopathological findings

To ensure that the resected nodules corresponded to nodules observed inCT scans, the radiologic and surgical procedures were conducted on the same day; CT-guided microcoils were inserted to mark both the nodule and the visceral pleural surface. Intra-operative fluoroscopy was then used to identify the microcoils, and thus the nodule to be resected. Two chest pathologists (F.X.J., with 12 years of experience, and Z.H., with 23 years of experience), who were blinded to all clinical information reviewed the pathological specimens independently and classified the lesions as atypical adenomatous hyperplasia, AIS, MIA, or IAC in accordance with the criteria described in the 2015 World Health Organization Classification of Lung Tumors [13, 14];Disagreements were resolved by consensus. They also classified the histological subtypes of IACs as lepidic predominant, acinar predominant, papillary predominant, micropapillary predominant, solid predominant, or invasive mucinous adenocarcinoma and then classified all tumors as pre-IAC (including AIS and MIA) or IAC.

### Statistical analysis

For continuous predictors, optimal cutoff values were determined via the maximal Youden index (sensitivity + specificity − 1). Receiver operating characteristic curves, and corresponding areas under the curves, sensitivities and specificities were presented for these dichotomized predictors using the optimal cutoffs.

Integer cutoffs can be more convenient in clinical practice; thus, approximate integer of optimal cutoffs were applied for maximal and mean diameters (both 10 mm), CT-determined density (− 600 HU), and age (55 years). Although our cutoff value for CEA was 2.19 μg/L, we used the upper limit of the normal range of 5 μg/L as our standard for stratification.

These dichotomized predictors were described using frequencies and percentages, differences between IAC groups were compared using the χ^2^ test. Some patients had multiple nodules, to assess relationships between clinicopathological factors and lung IAC, generalized estimating equation (GEE) was applied for the correlated data using with a logit link function and a binomial distribution. The working correlation structure was selected according to the quasi-likelihood under the independence model criterion minimum principle. In multivariable model 1, variables with *p* < 0.1 in univariable analysis were included. In multivariable model 2, all independent variables were included to assess the stability of the results. Lastly, a multicollinearity test was performed on all independent variables. All statistical analyses were performed with SPSS Statistics, version 17.0(SPSS Inc., Chicago, IL, USA), and *p* < 0.05 was deemed to indicate statistical significance.

## Results

### Patient characteristics, radiological features, and pathological features

**Fig. 1:**
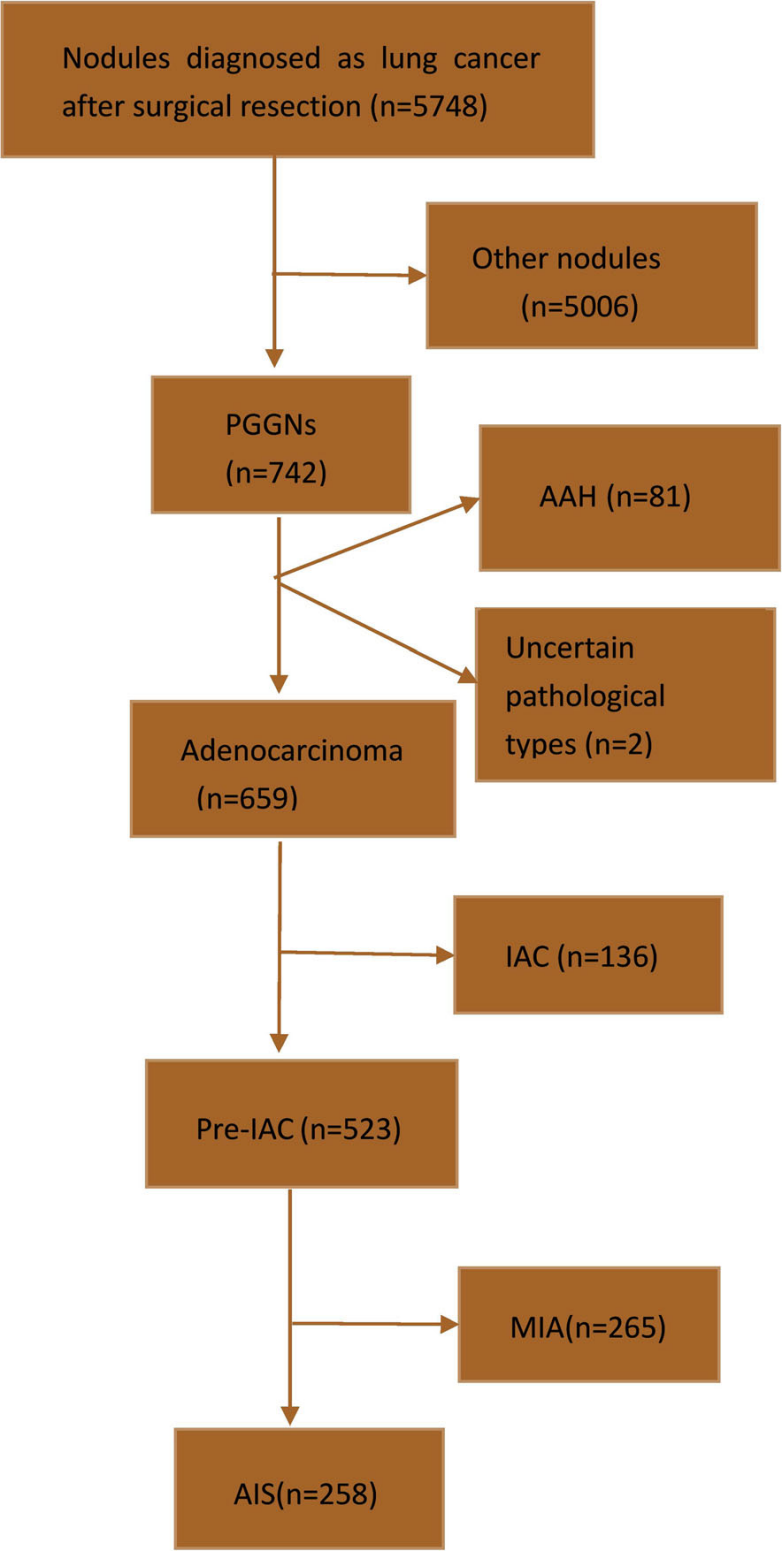
Flow chart of the classification process

In total, 659 pGGNs were detected in 641 patients, of whom 449 were women and 192 were men. The median age was 62.7 years (range 26–78 years). Eight men and 10 women had two pGGNs each. Of the 659 pGGNs 523 (79.4%) were pre-IACs, including 258 (39.2%) AISs and 265 (40.2%) MIAs. The remaining 136pGGNs (20.6%) were IACs, including 58 (8.8%) lepidic predominant, 33 (5.0%) acinar predominant, 43 (6.5%) papillary predominant, and two (0.3%) solid predominant adenocarcinomas. Figure 1 is a flow chart depicting the process ofnodule classification. There were no significant differences in CEA concentration (*p* = 0.792), or the frequencies of pleural retraction (*p* = 0.857), air bronchogram signs (*p* = 1.000), or bubble lucency signs (*p* = 0.598) between the pre-IAC and IAC groups. There were significant differences in the sex distribution (*p* = 0.025), age (*p* < 0.001), CT density (*p* < 0.001), spiculation signs (*p* = 0.021), and mean and maximal diameters (both *p* < 0.001) between these two groups. The relevant data are summarized in Table 1.

**Table 1:** Baseline patient characteristics by diagnostic category

Variable	Total (*n* = 659)	Pre-IAC (*n* = 523)	IAC (*n* = 136)	χ^2^	*p*
Sex				5.042	0.025
Male	200 (30.3%)	148 (28.3%)	52 (38.2%)		
Female	459 (69.7%)	375 (71.7%)	84 (61.8%)		
Age, years				40.540	< 0.001
≤ 55	325 (49.3%)	291 (55.6%)	34 (25.0%)		
> 55	334 (50.7%)	232 (44.4%)	102 (75.0%)		
Smoking history				4.985	0.723
Never	350 (53.1%)	283 (54.1%)	67 (49.3%)		
Current or former	309 (46.9%)	240 (45.9%)	69 (50.7%)		
Symptoms				5.742	0.831
Absent	480 (72.8%)	410 (78.4%)	70 (51.5%)		
Present	179 (27.2%)	113 (21.6%)	66 (48.5%)		
Serum CEA, μg/L				.069	0.792
< 5	652 (99.1%)	518 (99.2%)	134 (98.5%)		
≥ 5	6 (0.9%)	4 (0.8%)	2 (1.5%)		
Maximal diameter, cm				145.831	< 0.001
≤ 1	430 (65.2%)	401 (76.7%)	29 (21.3%)		
> 1	229 (34.8%)	122 (23.3%)	107 (78.7%)		
Mean diameter, cm				188.727	< 0.001
≤ 1	475 (72.0%)	441 (84.3%)	34 (25.0%)		
> 1	184 (28.0%)	82 (15.7%)	102 (75.0%)		
CT density, HU				21.374	< 0.001
≤ −600	344 (52.2%)	297 (56.8%)	47 (34.6%)		
> −600	315 (47.8%)	226 (43.2%)	89 (65.4%)		
Pleural retraction				0.033	0.857
Absent	633 (96.1%)	502 (96.0%)	131 (96.3%)		
Present	26 (3.9%)	21 (4.0%)	5 (3.7%)		
Air bronchogram sign				.000	1.00
Absent	647 (98.2%)	513 (98.1%)	134 (98.5%)		
Present	12 (1.8%)	10 (1.9%)	2 (1.5%)		
Bubble lucency sign				0.278	0.598
Absent	598 (90.7%)	473 (90.4%)	125 (91.9%)		
Present	61 (9.3%)	50 (9.6%)	11 (8.1%)		
Spiculated sign				5.329	0.021
Absent	560 (85.0%)	453 (86.6%)	107 (78.7%)		
Present	99 (15.0%)	70 (13.4%)	29 (21.3%)		

*CEA* carcinoembryonic antigen, *CT* computed tomography, *HU* Hounsfield units, *IAC* invasive adenocarcinoma

### Optimal cutoff values for continuous predictors

**Fig. 2:**
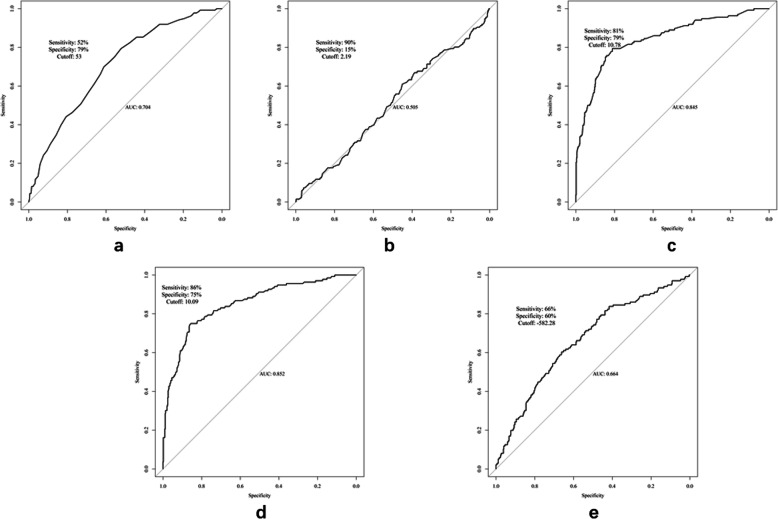
Receiver operating characteristic curves for age (**a**), serum carcinoembryonic antigen concentration (**b**), maximal diameter (**c**), mean diameter (**d**), and computed tomography-determined density (**e**). AUC, area under the receiver operating characteristic curve

The optimal cutoff values and areas under the curves for age, CEA, maximal and mean diameters, and CT-determined density for distinguishingbetween pre-IAC and IAC are summarized in Table 2. All data used to derive the sensitivity, specificity, positive predictive, and negative predictive values shown in Table 1 are provided as electronic supplementary material (ESM Table 1). Receiver operating characteristic curve analyses are presented in Fig. 2.

**Table 2:** Optimal cutoff values for continuous predictors

Indicator	AUC	p	Cut off value^**a**^	Sensitivity	Specificity	PPV	NPV
Age (years)	0.704 (0.657,0.751)	< 0.001	53	52%	79%	91%	30%
Serum CEA (μg/L)	0.495 (0.440,0.550)	0.862	2.19	90%	15%	80%	28%
Max diameter (mm)	0.845 (0.805,0.885)	< 0.001	10.78	81%	79%	94%	52%
CT value (HU)	0.664 (0.613,0.715)	< 0.001	−582.28	66%	60%	87%	32%
Mean diameter (mm)	0.852 (0.813,0.89)	< 0.001	10.09	86%	75%	93%	58%

^a^Cutoff values were determined via the maximal Youden index (sensitivity + specificity −1).*AUC* area under the receiver operating characteristic curve, *CEA* carcinoembryonic antigen, *CT* computed tomography, *HU* Hounsfield units, *Max* maximal, *NPV* negative predictive value, *PPV* positive predictive value

### Factors predicting IAC

**Fig. 3:**
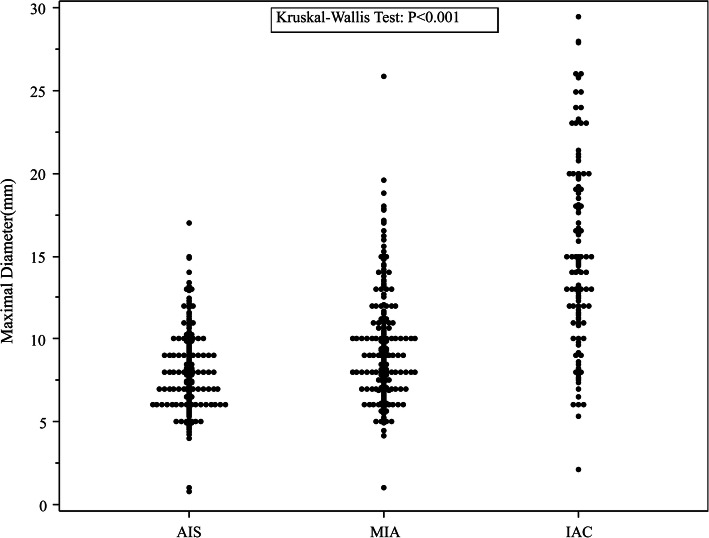
Scatter plot of pure ground-glass nodule maximal diameters plotted by pathological type (AIS, adenocarcinoma in situ; IAC, invasive adenocarcinoma; MIA, minimally invasive adenocarcinoma)

**Fig. 4:**
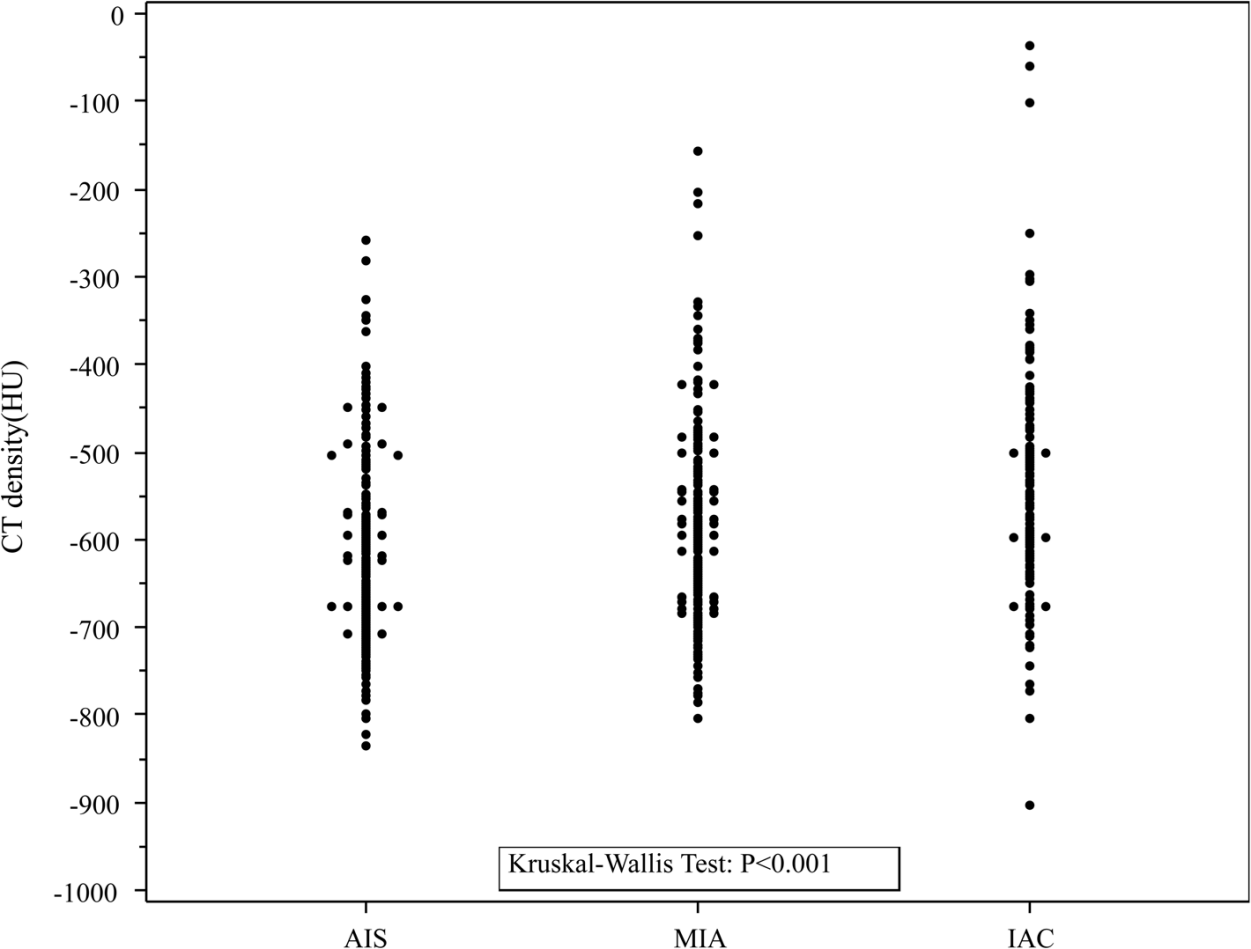
Scatter plot of computed tomography-determined density of pure ground-glass nodules plotted by pathological type (AIS, adenocarcinoma in situ; CT, computed tomography; HU, Hounsfield units; IAC, invasive adenocarcinoma; MIA, minimally invasive adenocarcinoma)

The results of the univariable and multivariable analyses are summarized in Table 3. In univariable analysis IAC was significantly associated with age (*p* < 0.001), sex (*p* = 0.024), mean and maximal diameters (both *p* < 0.001), CT density (*p* < 0.001), and spiculation signs (*p* = 0.02). Maximal nodule diameters are plotted by pathological type in Fig. 3. Of the 523 pre-IACs, 122 (23.3%) were > 10 mm in diameter. Of the 136 IACs, 107 (78.7%) were > 10 mm in diameter. With regard to CT density, 226 (43.2%) of the 523 pre-IACs exhibited CT densities > − 600 HU, and 89 (65.4%) of the 136 IACs exhibited CT densities > − 600 HU. Of the 229 pGGNs that were > 10 mm in diameter, 122 (53.3%) were pre-IACs (41 AISs and 81 MIAs) and 107 (46.7%) were IACs (Kruskal-Wallis test *p* < 0.001). pGGN CT densities are plotted by pathological type in Fig. 4. Of the 315 nodules with a CT density > − 600 HU, 226 (71.7%) were pre-IACs (87 AISs and 139 MIAs) and 89 (28.3%) were IACs (Kruskal-Wallis test *p* < 0.001).

**Table 3:** Univariable and multivariable analysis of associations between invasive adenocarcinoma and potential predictors thereof. Working correlation structure in the generalized estimation model was determined via quasi-likelihood under the independence model criterion, and unstructured working correlation structure was selected. Multivariable model 1 only included variables whose *p* values were < 0.1 in the univariable model. Multivariable model 2 included all potential variables, and was performed as a sensitivity analysis

		Univariable model		Multivariable Model 1		Multivariable Model 2	
Characteristics		OR (95% CI)	*p*	OR (95% CI)	*p*	OR (95% CI)	*p*
Sex	Female	0.634 (0.427, 0.942)	0.024	0.784 (0.468, 1.313)	0.356	0.708 (0.415, 1.208)	0.205
	Male	Reference		Reference		Reference	
Age, years	> 55	3.764 (2.464, 5.748)	< 0.001	2.240 (1.370, 3.665)	0.001	2.732 (1.605, 4.651)	< 0.001
	≤55	Reference		Reference		Reference	
Smoking history	Current or former	0.801 (0.590, 1.284)	0.722	–	–	0.721 (0.401, 1.023)	0.942
	Never	Reference		Reference		Reference	
Symptoms	Present	1.080 (0.780, 2.196)	0.830	–	–	0.988 (0.697, 2.010)	0.984
	Absent	Reference		Reference		Reference	
Serum CEA, μg/L	> 1	1.932 (0.350, 10.661)	0.45	–	–	0.967 (0.235, 3.978)	0.963
	≤1	Reference		Reference		Reference	
Maximal diameter, cm	> 1	13.058 (8.192, 20.814)	< 0.001	2.690 (0.994, 7.276)	0.051	2.992 (1.098, 8.153)	0.032
	≤1	Reference		Reference		Reference	
Mean diameter, cm	> 1	16.190 (10.285, 25.486)	< 0.001	6.207 (2.352, 16.383)	< 0.001	4.707 (1.761, 12.583)	0.002
	≤1	Reference		Reference		Reference	
CT density, HU	> − 600	2.612 (1.756, 3.885)	< 0.001	3.542 (2.155, 5.822)	< 0.001	3.886 (2.307, 6.548)	< 0.001
	≤ − 600	Reference		Reference		Reference	
Pleural retraction	Present	0.991 (0.329, 2.986)	0.988	–	–	2.042 (0.444, 9.390)	0.359
	Absent	Reference		Reference		Reference	
Air bronchogram sign	Present	0.765 (0.166, 3.535)	0.732	–	–	0.193 (0.042, 0.882)	0.034
	Absent	Reference		Reference		Reference	
Bubble lucency sign	Present	0.824 (0.415, 1.639)	0.582	–	–	0.583 (0.249, 1.365)	0.214
	Absent	Reference		Reference		Reference	
Spiculated sign	Present	1.761 (1.093, 2.838)	0.02	1.445 (0.781, 2.671)	0.241	1.758 (0.880, 3.511)	0.11
	Absent	Reference		Reference		Reference	

*CEA* carcinoembryonic antigen, *CI* confidence interval, *CT* computed tomography, *HU* Hounsfield units, *IAC* invasive adenocarcinoma, *OR* odds ratio (estimated from generalized estimation model)

In multivariable analysis, model 1 which included input variables that had *p* < 0.1 in univariable analysis (sex, age, maximal and mean diameters, CT density, and spiculation signs), age (*p* < 0.001), mean diameter (*p* = 0.002), and CT density (*p* < 0.001) were significant independent factors for differentiating pre-IACs and IACs (Table 3). In multivariable analysis, model 2 which included all potential variables and was conducted to assess sensitivity, age (*p* < 0.001), maximal diameter (*p* = 0.032), mean diameter (*p* = 0.002), and CT density (*p* < 0.001) were significant independent factors for distinguishing between pre-IACs and IACs (Table 3). A multicollinearity test indicated no multicollinearity problems in the multivariable models (Table 4).

**Table 4:** Multicollinearity test from multivariable models 1 and 2

Index	Criterion	Multivariable Model 1	Multivariable Model 2
Conditional index	< 10	6.52	6.48
Minimal tolerance	> 0.1	0.26	0.27
Maximal variance inflation factor	< 10	3.83	3.69

**Fig. 5:**
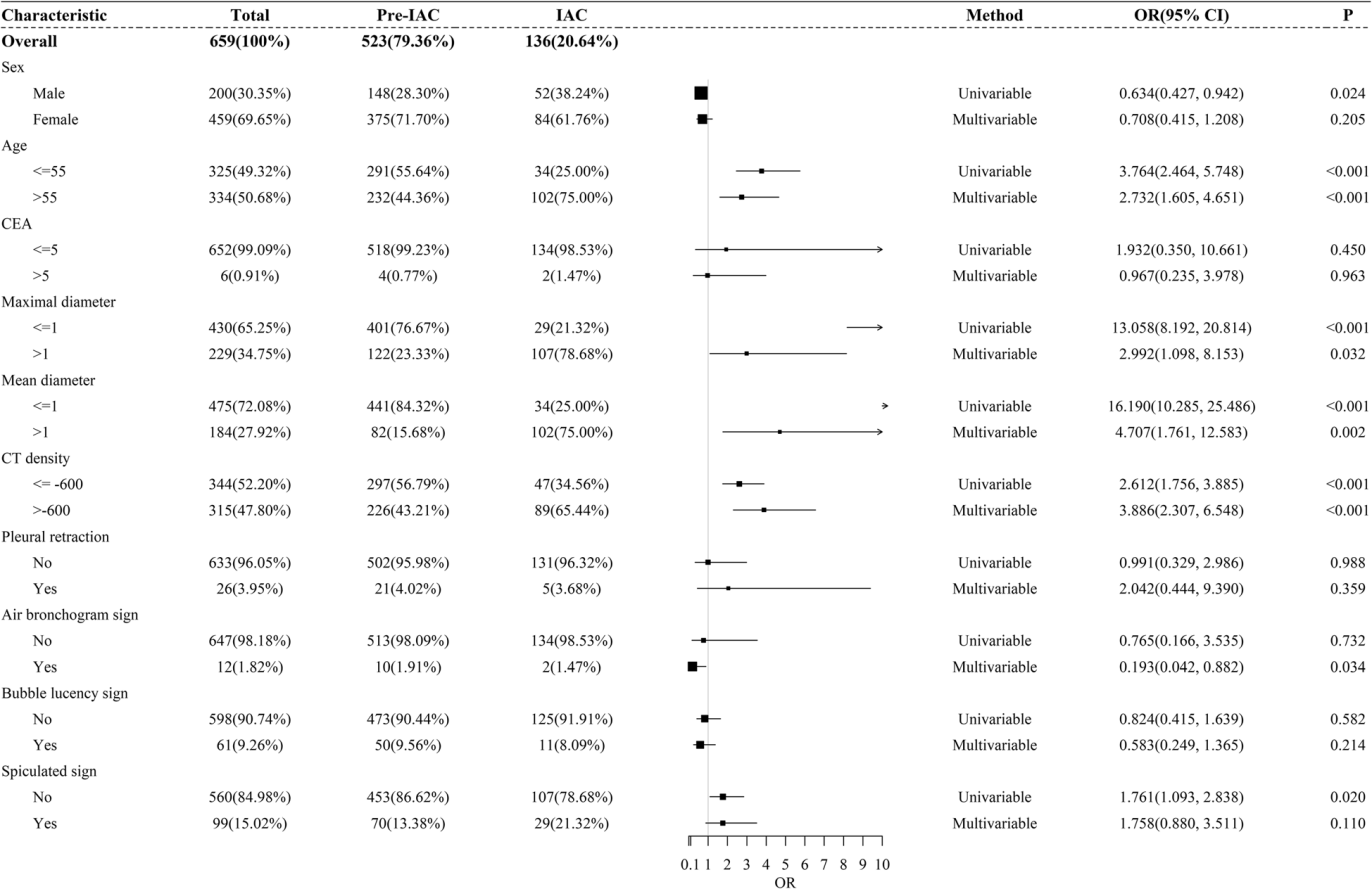
Forest plot of all results of univariable and multivariable analyses

All univariable and multivariable analysis results are presented as a forest plot in Fig. 5. Age, maximal diameter, mean diameter, and CT density were all independent factors that distinguished IAC from pre-IAC.

### Recurrence of pGGNs

After a median follow-up interval of 41 months (range 7–52 months) after resection, no pGGN IACs or pre-IACs had recurred. After resection, chest CT scans were periodically conducted and no new nodules were detected.

## Discussion

Several studies have analyzed relationships between CT features of pGGNs and pathological type [15–18]. However, to the best of our knowledge, no published study has identified predictors of lung IACs in pGGNs by analyzing their clinical and imaging characteristics. The present study investigated 659 adenocarcinomas manifesting as pGGNs on CT; thus, making the study was larger than most recent studies regarding pathological classification of pGGNs [11, 15, 17, 19, 20].

Kitami et al. [15] reported cutoffs of − 600 HU for CT density and 10 mm for maximum diameter for distinguishing between IAC and non-IAC pGGNs of the lung. The corresponding cutoffs identified in the current study are similar to those values, and other previously reported values [11]. Because integers are more convenient in clinical practice, we used published cutoff values in our univariable and multivariable analyses (age > 55 years, maximal diameter > 1 cm, CT density > − 600 HU). In the present study, model 2 suggested that maximal and mean diameters could reliably and independently distinguish IACs in patients with pGGNs with diameters of < 3 cm; this finding was consistent with reported literature [11, 15]. In the current study, a tumor diameter of > 10 mm was a significant independent predictor of IAC; however, that result is not readily apparent from the scatter plot shown in Fig. 3. The best predictor of IAC may be a diameter of > 20 mm, because only one of the pre-IACs shown in Fig. 3 had a diameter > 20 mm. Notably, that threshold yields a high positive predictive value; however, the corresponding negative predictive value would be abysmal. This would result in a high rate of missed diagnoses in clinical practice. Importantly, the observation in the present study that a tumor diameter of > 10 mm was an independent predictor of IAC is consistent with results reported in similar previous studies [17, 18, 21–23]. As depicted in the scatter plot shown in Fig. 3, a more aggressive histological subtype tended to be associated with larger pGGNdiameter. Thus, tumor size is the most important variable on which to base decisions pertaining to the management of pGGNs [12, 24, 25].

Although nodules > 10 mm in diameter are more likely to be IACs, smaller nodules may also be IACs. In the present study 29 nodules with diameters of < 10 mm were found to be IACs. Persistent nodules larger than 5 mm should be followed for at least 4 years. And growth of more than 2 mm in maximal diameter is considered significant [26]. It is appropriate to monitor nodules with diameters < 10 mm independently. With regard to these smaller nodules, further research is needed to establish a basis for clinical planning.

Although CT-determined density of pGGNs of > − 600 HU was a significant predictor of IAC in the present study, the scatter plot shown in Fig. 4 does not clearly depict this result. We presume that the use ofa single factor to predict IACs is not good clinical practice; simultaneous assessment of multiple factors would be more accurate. Notably, Kitami et al. [15] reported that the CT-determined density of pGGNs can distinguish IACs, and Lim et al. [11] reported that pGGN density was a significant predictor of tumor invasiveness. In contrast, Heidinger et al. [17] reported that pGGN density was not significantly associated with pathological diagnosis, and several other groups have also reported no significant differences in nodule density between AISs, MIAs, and IACs manifesting as pGGNs on CT [20, 21]. All of these studies were carried out with similar sampling methods. Thus, whether CT density is a valid parameter for distinguishing IACs remains controversial. CT density should be combined with other indicators such as size, patient age, and certain CT signs when predicting the nature of a lesion preoperatively.

Liu et al. [22] reported that the presence of signs of spiculation is suggestive of a diagnosis of IAC. In univariable analysis in the present study spiculation was a significant predictor of IAC; however, this was not confirmed in multivariable analysis. Spiculation is considered to be evidence of malignancy and to represent invasiveness. In one study spiculation was the strongest predictor of invasion [27].

In the present study age was a significant predictor of IAC. To the best of our knowledge no previous studies have identified this correlation. This hitherto unreported result of the current study warrants further investigation in prospective studies. In clinical practice, the recommendation of surgical resection in patients with pGGN who are aged > 55 years is determined with respect to a combination of other additional factors such as nodule diameter and nodule density. IACs presenting as pGGNs have a good prognosis. In the present study, no pGGN IACs recurred after surgical resection during a median follow-up interval of 41 months (range, 7–52 months); this finding is consistent with the results of previous investigations [28–30] .

IACs and pre-IACs are known to require treatment involving different surgical procedures. The risk of lung cancer is high if the nonsolid nodule size is greater than 8 mm [31]. At our hospital surgery is performed if the diameter of the pGGN is > 8 mm. Most patients are treated via limited resection and frozen section, and lobectomy is performed if an invasive component is detected via frozen section. Unfortunately, the accuracy of frozen section is not satisfactory [32]. Moreover, distinction between MIA and IAC remains difficult, regardless of the presence of invasive components. Because the accuracy of intraoperative frozen sections is not entirelyclear, it is easier to determine the appropriate treatment if IAC can be predicted accurately. Although limited resection is the preferred treatment for pGGN, it may not be possible to remove all lesions. In the present study 20.6% of pGGNs were eventually diagnosed as IACs, therefore the presence of a pGGN should not be used as an indication for limited resection.

The current study had some limitations. First, it was retrospective, and all the data were derived from a single institution. However, all data in the study were collected during 2016 and all patients were managed in accordance with the same protocol; thus, there was conceivably relatively minimal bias. Second, this study only included patients in whom a diagnosis had been established via resection, whereas some unresected pGGNs may also have been adenocarcinomas. These factors may have contributed to a selection bias in the present study. A prospective study would be required to minimize these potential sources of bias. Third, distinguishing between pGGNs and other nodules is subjective in this study. Two reviewers (a radiologist and a surgeon) evaluated all CT scans independently to minimize this source of bias. Finally, blood vessels and bronchioles could not always be fully excluded when the boundaries of lesions were delineated, this may have contributed to variations between observers in nodule measurements and lesion characterization.

## Conclusions

Patients with pGGNs < 3 cm in diameter on CT are more likely to have IACs if they are aged > 55 years, exhibit a nodule diameter > 1 cm, and have a CT-determined nodule density > − 600 HU. Postoperatively, IACs initially identified as pGGNs have a good prognosis, and there were no recurrences during a median follow-up interval of 41 months in the current study. The results of this study may assist decisions pertaining to the selection of surgical procedures in patients with pGGNs identified as being at high risk of malignant disease.

## Supplementary information

**Additional file 1.**

## Data Availability

The datasets used and/or analysed during the current study are available from the corresponding author on reasonable request.

## Abbreviations

pGGNs: Pure ground-glass nodules
CT: Computed tomography
IACs: Invasive adenocarcinoma
HU: Hounsfield units
AIS: Adenocarcinoma in situ
MIA: Minimally invasive adenocarcinoma
CEA: Serum carcinoembryonic antigen
