# Supplementary material for: Factors distinguishing invasive from pre-invasive adenocarcinoma presenting as pure ground glass pulmonary nodules
Source: Radiat Oncol. 2020 Jul 31;15:186. doi: 10.1186/s13014-020-01628-x (PMC7393870; doi:10.1186/s13014-020-01628-x)
Supplement: Supplementary file 1 — Additional file 1. [file 13014_2020_1628_MOESM1_ESM.docx]

| **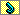indicator** | **area_CI** | **p** | **True positive** | **True negative** | **False positive** | **False negative** | **cutoff** | **sensitivity** | **specificity** | **PPV** | **NPV** |
| --- | --- | --- | --- | --- | --- | --- | --- | --- | --- | --- | --- |
| Age | 0.704(0.657 ,0.751) | <0.001 | 273 | 108 | 28 | 250 | 53 | 0.522 | 0.794 | 0.90698 | 0.30168 |
| CEA | 0.495(0.44 ,0.55) | 0.862 | 467 | 21 | 115 | 55 | 2.19 | 0.895 | 0.154 | 0.80241 | 0.27632 |
| Maicimal diameter | 0.845(0.805 ,0.885) | <0.001 | 424 | 108 | 28 | 99 | 10.78 | 0.811 | 0.794 | 0.93805 | 0.52174 |
| CT density | 0.664(0.613 ,0.715) | <0.001 | 346 | 82 | 54 | 177 | -582.28 | 0.662 | 0.603 | 0.865 | 0.3166 |
| Mean diameter | 0.852(0.813 ,0.89) | <0.001 | 448 | 102 | 34 | 75 | 10.09 | 0.857 | 0.75 | 0.92946 | 0.57627 |

ESM Table1
